# Isolation and genomic characterization of S*taphylococcus aureus* bacteriophages from Chennai, India

**DOI:** 10.1128/mra.01209-23

**Published:** 2024-03-08

**Authors:** Shankaregowdanakopalu Jagadeesh Deepak, Porteen Kannan, Wilfred Ruban Savariraj, Anbazhagan S, Elango Ayyasamy, Senthil Kumar Tuticorin Maragatham Alagesan, Narendra Babu Ravindran, Sureshkannan Sundaram, Nithya Quintoil Mohanadasse, Teresa D. Shippy, Charley A. Cull, Gizem Levent, Raghavendra G. Amachawadi

**Affiliations:** 1 Department of Veterinary Public Health and Epidemiology, Madras Veterinary College, TANUVAS, Chennai, India; 2 Department of Livestock Products and Technology, Veterinary College, KVAFSU, Bengaluru, India; 3 ICMR-National Animal Resource Facility for Biomedical Research (NARFBR), Hyderabad, Telangana, India; 4 Veterinary College and Research Institute, TANUVAS, Salem, India; 5 Zoonoses Research Laboratory, Centre for Animal Health Studies, TANUVAS, Chennai, India; 6 Department of Livestock Products and Technology, Madras Veterinary College, TANUVAS, Chennai, India; 7 Department of Veterinary Public Health and Epidemiology, RIVER, Pondicherry, India; 8 Bioinformatics Center, Division of Biology, Kansas State University, Manhattan, Kansas, USA; 9 Midwest Veterinary Services, Inc., Oakland, Nebraska, USA; 10 Texas Tech University School of Veterinary Medicine, Amarillo, Texas, USA; 11 Department of Clinical Sciences, College of Veterinary Medicine, Kansas State University, Manhattan, Kansas, USA; Portland State University, Portland, Oregon, USA

**Keywords:** Caudoviricetes, lytic bacteriophages, DNA sequencing

## Abstract

We isolated and characterized two lytic bacteriophages against *Staphylococcus aureus* named TANUVAS_MVC-VPHSA1 and TANUVAS_MVC-VPHSA2, with the aim of investigating their genomic and structural features. The bacteriophages belong to the Caudoviricetes, and their genomes have sizes of 50,505 and 50,516 base pairs with a GC content of 41.4%.

## ANNOUNCEMENT

The increased antimicrobial resistance in *Staphylococcus aureus* (1) and the Food and Drug Adminstration (FDA) (2) stringent regulations on the use of antimicrobials in food led the search for safer alternatives. The bacteriophages have emerged as promising candidates due to their specificity (3) and their potential applications in food safety and therapy (4, 5).

**Fig 1:**
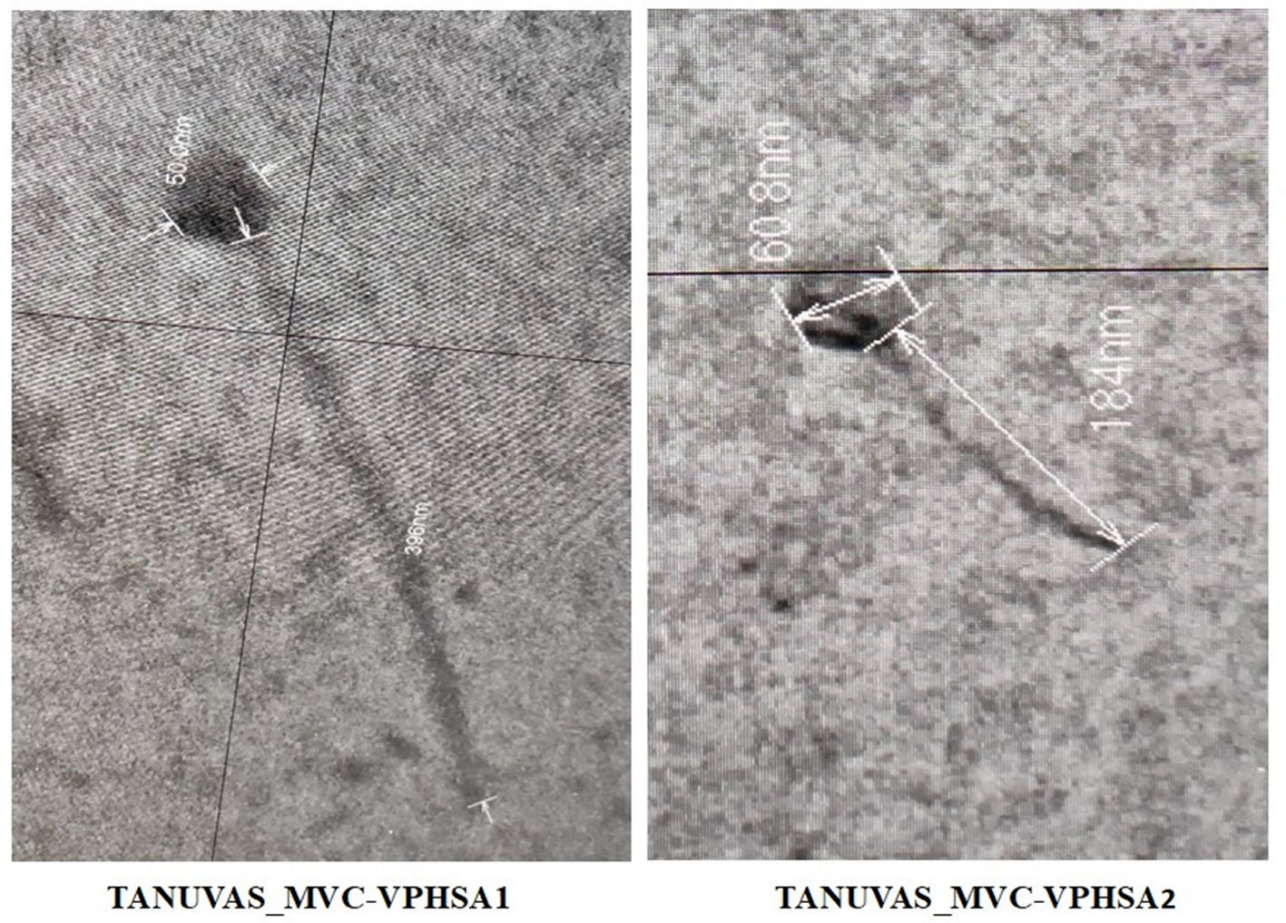
Transmission electron microscopic image of bacteriophages showing icosahedral-shaped heads and non-contractile, flexible tails against *S. aureus* from Chennai, India (TANUVAS_MVC-VPHSA1 and TANUVAS_MVC-VPHSA2).

We isolated the bacteriophages from effluent waters of treatment plants in hospitals and slaughterhouses, Chennai, India (13.0827° N, 80.2707° E). The 50-mL effluent water was left undisturbed overnight. The 10 mL supernatant in 40 mL 5× LB broth, and exponentially grown culture of *Staphylococcus aureus* strain ATCC 25923 was incubated overnight at 37°C using shaker incubator. Later, *Staphylococcus aureus* strain ATCC 25923 was centrifugated at 7,000 *g*, 10 min at 4°C, and supernatant was collected and filtered through a pre-sterilized 0.45/0.22-µm PVDF filter and plated using the double agar overlay method against *Staphylococcus aureus* (6). A single irregular corrugated (TANUVAS_MVC-VPHSA1) and clear round (TANUVAS_MVC-VPHSA2) plaque was picked and passaged twice on double agar overlay. Phages were precipitated from lysates with PEG/NaCl and resuspended with SM buffer (6). For transmission electron microscopy, precipitated phage suspensions were coated on carbon-coated formvar grids and stained with phosphotungstic acid (6) and images were captured (*n* = 5 particle) (HITACHI Ltd., HT7700, Japan) at 80 kV (Fig. 1).

The DNA was extracted using the phenol-chloroform method (7). The DNA library and barcoding were prepared with end-repair/dA tailing using Ultra II End prep enzyme mix (NEBNext Ultra II, NEB) and DNA barcode adapter (2X ligation MasterMix, NEB); finally, barcode-ligated fragments were purified using AMPureXP beads. The genome sequencing with MinION sequencer coupled SpotON flow cell, where 75 µL of pooled library (430 ng) was added through SpotON sample port. Using the MinKNOW interface, sequencing was run in FAST5 mode for 48 hr and basecalling was performed with Guppy 6.1.1. Canu v1.9 (8) which was employed for assembly with a genome size set to 50 kb and stopOnReadQuality = false and BLAST+ v2.14 (9) was used for genome comparisons. Assembly of selected reads (longer than 1,000 bp with overlaps) produced single contigs for each genome (see Table 1 for statistics). The tRNA prediction was done with tRNAscan-SE 2.0 (10), and genomes were annotated using Pharokka v1.4.1 (11). Predicted genes were compared with PHROGS (12), VFDB (13), and CARD (14) using MMseqs2 (15) and PyHMMER (16) within Pharokka (11). A few predicted genes that did not meet GenBank criteria were removed from the annotation during the submission process. All tools were run with default parameters unless otherwise mentioned.

**TABLE 1:** The structural and genomic sequencing summary and characteristics of bacteriophages against *S. aureus* from Chennai, India^*a*^

Bacteriophage name	TANUVAS_MVC-VPHSA1	TANUVAS_MVC-VPHSA2
Sample origin	Hospital treatment plant	Slaughter house treatment plant
Tail length	375–410 nm	175–198 nm
Head diameter	50–55nm	60–65nm
Length (bp)	50,505 bp	50,516 bp
GC content (%)	41.4%	41.4%
Total reads	1694	2246
Reads > 1,000 bp with overlaps	597	978
Read N50	3,428 bp	3,424 bp
Coverage	33.96×	55.08×
No. of CDS (GenBank)	109	92
No. of genes with predicted function (Pharokka)	49	43
Genes with unknown function (Pharokka)	63	50
No. of tRNAs and tmRNAs	0	0
Head and packaging genes	11	10
DNA, RNA, and nucleotide metabolism genes	21	16
Connector genes	2	2
Moron, auxiliary metabolic gene and host takeover, and tail	12	11
Temperate marker genes (integration and excision)	0	0
Transcription regulation genes	1	2
Lytic genes	2	2
Antibiotic resistance genes	0	0
Virulence genes	0	0
CRISPRs	0	0

^*a*^ (TANUVAS_MVC-VPHSA1 and TANUVAS_MVC-VPHSA2).

The phages exhibited direct terminal repeats in their double-stranded DNA genomes. MegaBLAST (17) alignment showed 99.90% nucleotide identity between TANUVAS_MVC-VPHSA1 and TANUVAS_MVC-VPHSA2. Functional annotation revealed putative endolysin genes [OR670591.1 (FBHYGVHD_CDS0041) and OR670592.1 (YTCETSXE_CDS0086)], suggesting for bacterial lysis. BLASTp against the NCBI nr databases indicated that the putative endolysin protein sequences share 72% identity and 100% coverage with the N-acetylmuramoyl-L-alanine amidase (WNL49492) of Bacillus phage DZ1. BLASTn (9) against the GenBank nr/nt database identified *Bacillus cereus* DZ1 phage as the closest genome match with 90% query coverage and 90% identity, consistent with the assignment of these genomes to the Caudoviricetes.

## Data Availability

The genome sequences and associated data of *Staphylococcus aureus* 86 phages TANUVAS_MVC-VPHSA1 and TANUVAS_MVC-VPHSA2 were deposited in 87 NCBI under the BioProject accession number PRJNA1025853, Genome accession numbers 88 OR670591 and OR670592, and SRA accession numbers SRR26351516 and SRR26351517.
